# External beam radiotherapy with or without californium-252 neutron brachytherapy for treatment of recurrence after definitive chemoradiotherapy

**DOI:** 10.1038/s41598-020-78074-y

**Published:** 2020-12-01

**Authors:** Wen-an Wu, Yi-ping Yang, Jing Liang, Jin Zhao, Jian-sheng Wang, Jia Zhang

**Affiliations:** 1 The First Affiliated Hospital of Xi’an Jiao Tong University, Xi’an, 710061 China; 2 The Shaanxi Provincial Tumor Hospital, Xi’an, 710061 China

**Keywords:** Radiotherapy, Cancer

## Abstract

We aimed to evaluate the application of external beam radiotherapy (EBRT) combined with californium-252 (^252^Cf) neutron intraluminal brachytherapy (NBT) in patients with local recurrent esophageal cancer after definitive chemoradiotherapy (CRT). Sixty-two patients with local recurrent esophageal squamous cell carcinoma after definitive CRT were retrospectively analyzed; 31 patients underwent NBT+EBRT, and 31 received EBRT alone. The response rate; 1-, 2-, and 3-year overall survival rates; and adverse event occurrence rates were compared between these two patient groups. The response rate was 83.87% (26/31) in the NBT+EBRT group and 67.74% (21/31) in the EBRT group (*p* < 0.001). The 1-, 2-, and 3-year overall survival rates were 80.6%, 32.3%, and 6.5%, respectively, in the EBRT group, with a median survival time of 18 months. The 1-, 2-, and 3-year overall survival rates were 83.8%, 41.9%, and 6.9%, respectively, in the NBT+EBRT group, with a median survival time of 19 months. The differences between the groups were not significant (*p* = 0.352). Regarding acute toxicity, no incidences of fistula or massive bleeding were observed during the treatment period. The incidences of severe and late complications were not significantly different between the two groups (*p* = 0.080). However, the causes of death for all patients differed between the groups. Our data indicate that ^252^Cf-NBT+EBRT produces favorable local control for patients with local recurrent esophageal cancer after CRT, with tolerable side effects.

## Introduction

In 2014, approximately 200,000 new patients in China were diagnosed with local recurrent esophageal cancer^1^, and the prevalence of this disease is expected to be younger and increase. Squamous cell carcinoma is the predominant histological subtype and likely develops in the middle and upper thoracic esophagus^2,3^. Esophagectomy is the standard treatment strategy for localized esophageal cancer. For advanced esophageal carcinoma or patients who decline or cannot tolerate surgery, definitive radiotherapy (RT) is an alternative^4^.

The most common treatment failure for esophageal cancer after definitive RT or chemoradiotherapy (CRT) is local recurrence^5^. The recurrence rate after definitive RT, CRT and/or surgery reaches 70%, and the 5-year survival rate is also low^6^. Local recurrence in the radiation field is the most important reason for radiotherapy failure. Furthermore, most patients with recurrence are no longer eligible for surgery. Thus, repeated irradiation is required for some right patients. The advantage of RT as a primary treatment for esophageal carcinoma or its local recurrence has been widely demonstrated. However, the prescription dose to reirradiate a target is limited by dose constraints of the surrounding normal tissue, and the local recurrence of esophageal carcinoma after definitive RT or chemoradiotherapy (CRT) is resistant to conventional photon radiotherapy. Brachytherapy is appropriate for the local recurrence of esophageal cancer after RT/CRT due to the high doses of radiation to the tumor and the low doses to nearby normal tissues^7^. Evidence from randomized trials has revealed that the outcome of single-fraction intraluminal brachytherapy is better than that of stents, in which intraluminal brachytherapy improves dysphagia and quality of life associated with the delayed onset of symptomatic relief^8^.

On the other hand, californium-252 (^252^Cf) neutron intraluminal brachytherapy (NBT) is a high linear energy transfer RT approach effective for treating radioresistant cancer and intracavitary cancers of the cervix, colon/rectum and esophagus when combined with external beam RT (EBRT)^9^.

To date, no multicenter, randomized prospective trials have compared the efficacy of NBT+EBRT and EBRT for the treatment of esophageal carcinoma and recurrence after definitive CRT. Accordingly, we conducted a retrospective study to evaluate the morbidity and effectiveness of EBRT combined with ^252^Cf-NBT in treating patients with local recurrent esophageal carcinoma after definitive CRT.

## Materials and methods

### General clinical data

The present study was approved by the Protection of Human Subjects Committee of Shaanxi Provincial Tumor Hospital (No. 2010–007) and complied with the Declaration of Helsinki. Informed consent was obtained from all patients.

A total of 62 patients with local recurrent esophageal carcinoma received definitive CRT between August 2010 and August 2018. The inclusion criteria were as follows: (1) patients who received definitive CRT as the initial treatment for esophageal cancer, (2) patients with squamous cell carcinoma, (3) patients with pathologically confirmed local recurrence without local or distal lymph node recurrence, (4) no salvage esophagectomy after recurrence, (5) no serious medical history or illness, and (6) no perforation of the esophagus or deep ulcer of the esophagus. All patients were divided into two groups based on the treatment received: the NBT+EBRT group, patients who received EBRT combined with ^252^Cf-NBT; and the EBRT group, patients who received only EBRT. The clinical profiles and manifestations of all patients are summarized in Table 1.

**Table 1:** Patients characteristics (N = 62).

Characteristics	EBRT (N)	NBT+EBRT (N)	*p *value
**Sex**			0.758
Male	18	17	
Female	13	14	
**Age (years)**			1.095
≤ 50	16	14	
> 50	15	17	
**Location of tumor**			0.936
Upper	12	14	
Middle	16	12	
Lower	3	5	
**Initial length**			0.625
≤ 5 cm	18	17	
> 5 cm	13	14	
**Pathological grade**			0.085
I	5	7	
II	20	19	
III	6	5	
**Initial clinical stage**			0.785
II	14	12	
III	17	19	
**Initial ECOG-PS**			0.634
0–1	20	19	
2	11	12	
3	0	0	
**Initial radiation dose(GY)**			0.082
≥ 60 Gy	28	29	
< 60 Gy	3	2	
**RFS**			0.093
≥ 12 months	19	20	
< 12 months	12	11	

*EBRT* external beam radiotherapy, *ECOG-PS* Eastern Cooperative Oncology Group performance status, *NBT* neutron intraluminal brachytherapy, *RFS *recurrence-free survival.

### Treatment

#### Group: EBRT

Intensity-modulated RT was delivered to patients in the EBRT group. First, simulation CT scanning was performed, and images were transferred to a planning system. Second, physicians delineated the gross tumor volume (GTV) utilizing information from an endoscopic investigation and magnetic resonance imaging (MRI). Margins of 0.8 cm and 1.0 cm along the superior and inferior directions were given to the GTV for the clinical target volume (CTV). A margin of 0.5 cm in all directions was given to the CTV for the planning target volume (PTV). A dose of 54–60 Gy was prescribed for salvage RT, with 1.8–2 Gy/f for 5 f./w (30 fractions total).

Group: EBRT+NBT.

During the treatment period, EBRT was carried out with intensity-modulated RT. The GTV was determined according to the endoscopic investigation and magnetic resonance imaging (MRI). The total dose via EBRT was 41.4–46 Gy, four fractions/week, one fraction/day, and 1.8–2.0 Gy/fraction, for a total of 23 fractions.

NBT was implemented with a ^252^Cf-based LZH-1000 intracavitary radiotherapy machine (Shenzhen, China). Californium-252 has a half-life of 2.65 years and, on decay, emits 2.31 × 10^6^ neutrons/s/µg and 1.32 × 10^7^ gamma photons/s/µg. The mean neutron energy was 2.2 meV, and the mean energy of gamma photons was 0.8 meV. Liu et al^10^. described the relative biological effectiveness (RBE) value of neutrons. Both the RBE value of the neutron and algebraic formula were included in the treatment plan of the NBT system. The source applicator includes a water balloon surrounding the source. The water balloon is 12 cm long, and the diameter can vary. The water balloon is an important part of the source applicator because it can keep the source close to the tumor but away from the adjacent normal epithelium. The position of the source capsule was determined on the X-ray image and then used as input for the treatment system. The reference point of the prescription for NBT was on the transverse plane 10 mm from the center point of the source capsule. The total dose via NBT was 12Gy12 Gy-eq/3 f., and 4 Gy-eq/1 f./1 w.

### Organs at risk (OARs)

The dose limit was 45 Gy for initial CRT in the spinal cord and 20 Gy for salvage RT. The volume fractions of 5 Gy (V5) and 20 Gy (V20) in the lungs were restricted to 60% and 28%, respectively, for initial CRT and 55% and 25%, respectively, for recurrence therapy.

### Chemotherapy

Concurrent chemotherapy was recommended for all patients with local recurrent esophageal cancer after radical chemoradiotherapy. Adjuvant or induction chemotherapy was not recommended. All patients completed two cycles of S1 concurrent chemotherapy.

### Toxicity assessment and follow-up

Weekly blood tests and other examinations were performed throughout the course of treatment. We recorded all treatment-related complications. The adverse events were graded according to the National Cancer Institute Common Terminology Criteria for Adverse Events (version 3.0). Upon the completion of treatment, follow-up examinations were performed every 3–6 months. Repeated CT, barium swallow fluoroscopy and endoscopy were performed to evaluate the tumor responses and nodal diseases.

### Statistical analysis

All statistical analyses were performed using SPSS software (version 20.0). The continuous and categorical variables of these two groups were compared to baseline characteristics using *t*-tests and chi-square tests. Overall survival (OS) was defined as the time from the receipt of treatment to death or the last follow-up. Recurrence-free survival (RFS) was defined as the time from the receipt of treatment to the pathological confirmation of recurrence. Last, after-recurrence survival (ARS) was defined as the time from the pathological confirmation of recurrence to death. The Kaplan–Meier method was used to generate survival curves, and the curves were compared using log-rank tests. Factors that influenced survival were determined using Cox’s proportional hazards regression model. *P* < 0.05 was considered statistically significant.

## Results

### Responses to treatment

All patients exhibited a response to treatment. In the NBT+EBRT group, 84% of patients achieved complete response (CR) or partial response (PR), while the remaining 16% of patients achieved stable disease (SD). Furthermore, patients in the NBT+EBRT group exhibited significantly better responses than those in the EBRT group.

### Patient survival

The follow-up period ranged within 6–72 months, with a median follow-up of 23 months that ended in August 2018. During the follow-up period, all patients died. For the entire study population, OS ranged from 6–49 months, with a median OS duration of 19 months, and the 1-, 2- and 3-year OS rates were 83.8%, 37% and 8%, respectively. Moreover, the median RFS duration was 12 months, while the median ARS duration was 11 months.

**Figure 1:**
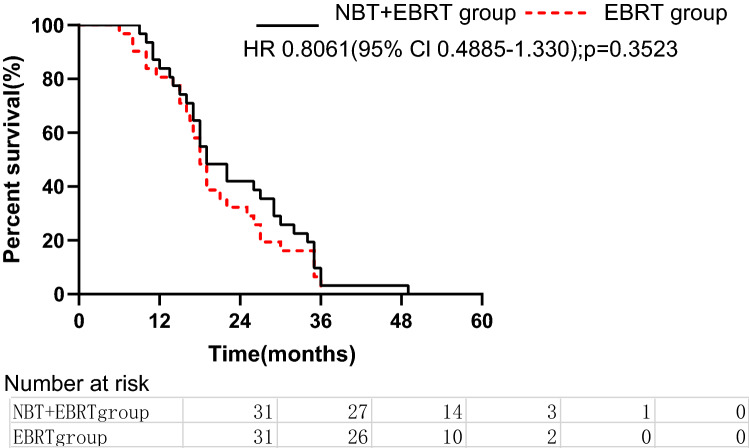
Overall survival curves of patients treated with external beam radiotherapy (EBRT) versus those who received neutron intraluminal brachytherapy (NBT) plus EBRT (log-rank *p* = 0.352).

For patients in the EBRT group, the 1-, 2- and 3-year OS rates were 80.6%, 32.3% and 6.5%, respectively, and the median OS duration was 18 months. For patients in the NBT+EBRT group, the 1-, 2- and 3-year OS rates were 83.8%, 41.9%, and 6.9%, respectively (Fig. 1), and the median OS duration was 19 months. There was no significant difference in the median OS duration between these two groups (*P* = 0.352).

**Figure 2:**
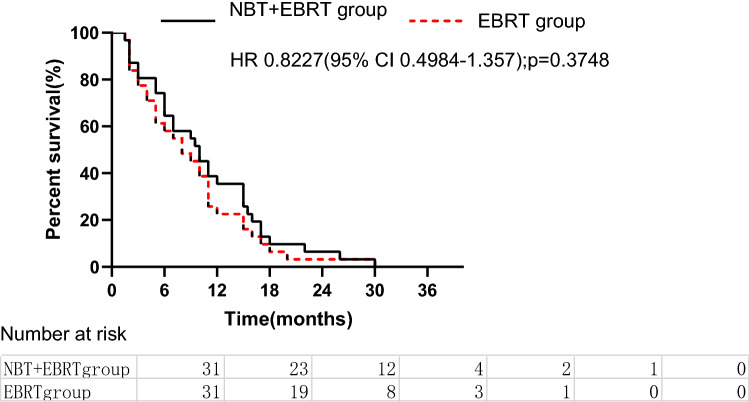
After-recurrence survival in patients who received external beam radiotherapy (EBRT) versus those who received neutron intraluminal brachytherapy (NBT) plus EBRT (log-rank *p* = 0.374).

The 6-month and 1-year ARS rates in the NBT+EBRT group were 74.2% and 61.3%, respectively. On the other hand, the 6-month and 1-year ARS rates in the EBRT group were 38.7% and 25.8%, respectively. There was no significant difference between these two groups (*P* = 0.374, Fig. 2).

Though not statistically significant (p = 0.667 with a 95% confidence interval), the median FRS duration for the NBT+EBRT group was 13 months (10.2 to 13.9 months), whereas that for the EBRT alone group was 12 months (10.6 to 14.6 months). These mean values suggest that EBRT alone is nonsignificantly superior to NBT+EBRT.

**Figure 3:**
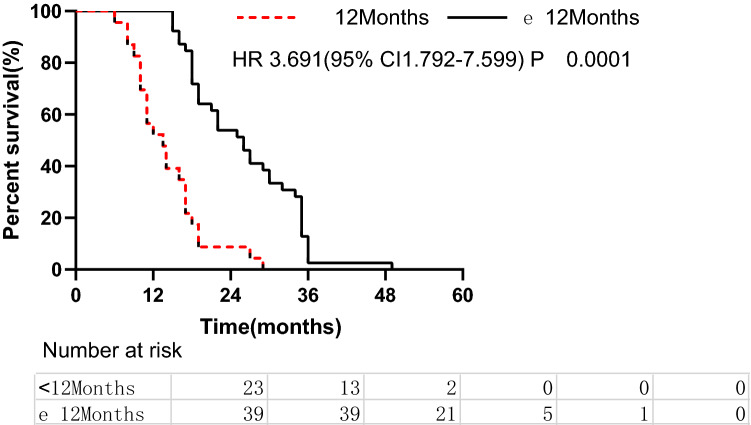
Overall survival of patients with late recurrence (≥ 12 months) versus those with early recurrence (< 12 months) (log-rank *p* < 0.001).

For patients whose recurrence occurred at ≥ 12 months, the median OS duration was 26 months, while the median OS duration for early recurrence (< 12 months) was 13.5 months (*P* < 0.001, Fig. 3). Furthermore, the median ARS duration for late recurrence was 10 months, while the median ARS duration for early recurrence was six months, and the difference was marginally significant (*P* = 0.053). The multivariate analysis of factors revealed that salvage RT and RFS are significant predictors of OS (Table 2).

**Table 2:** Prognostic factors evaluated by log-rank test survival analysis.

Characteristics	Number	Median OS (months)	Log-rank test	Univariate
			*p* value	*p* value
**Sex**			0.625	0.765
Male	35	18		
Female	27	17		
**Age (years)**			0.752	0.654
≤ 50	30	20		
> 50	32	19		
**Tumor location**			0.156	0.235
Upper	26	15		
Middle	28	17		
Lower	8	19		
**Initial length**			0.023	0.164
≤ 5 cm	35	21		
> 5 cm	27	16		
**Pathological grade**			0.102	0.274
I	12	20		
II	39	18		
III	11	15		
**Initial clinical stage**			0.412	0.657
II	26	20		
III	36	17		
**Initial ECOG-PS**			0.758	0.458
0–1	39	19		
2	23	18		
3	0			
**Initial radiation dose**			0.532	0.178
≥ 60 Gy	57	19		
< 60 Gy	5	18		
**RFS**			0.001	0.001
≥ 12 month	39	26		
< 12 month	23	13.5		
**Group**			0.352	0.473
NBT+EBRT	31	19		
EBRT	31	18		

*EBRT* external beam radiotherapy, *ECOG-PS* Eastern Cooperative Oncology Group performance status, *NBT* neutron intraluminal brachytherapy, *RFS* recurrence-free survival.

Table 3 shows the detailed death data of all patients at the end of follow-up. Regarding the causes of death in all patients, the difference between the two groups was statistically significant (*P* < 0.001).

**Table 3:** Causes of death (N = 62).

Cause of death	NBT+EBRT, N (%)	EBRT N (%)	*p* value
Local failure	3 (9.7)	5 (16)	0.001
Fistulas/massive bleeding	5 (16.3)	4 (12.9)	
Metastasis	22 (71)	15 (48.4)	
Lung infection	1 (3.2)	7 (22.6)	

*EBRT* external beam radiotherapy, *NBT* neutron intraluminal brachytherapy.

### Treatment-related toxicity

NBT and/or EBRT were completed by all patients. Throughout the treatment period, no perforations or massive bleeding was observed. Among these patients, 27 (43.5%) developed grade 2 hematologic toxicities, while 40 (64.5%) were diagnosed with grade 2 or higher esophagitis, as expressed by clinical odynophagia. After four to six weeks of treatment, three-quarters of patients resumed normal swallowing, while merely 3.6% of patients had residual dysphagia that required intermittent dilatation. Two patients (3.2%) had grade 2 or above irradiation dermatitis. During the follow-up period, four patients (6.5%) suffered from fistulas, and five patients (8.1%) had massive bleeding upon local recurrence. Moreover, four patients (12.9%) in the EBRT group exhibited radiation pneumonitis higher than grade 3, and all died of severe lung infection. Because patients experienced short survival durations after recurrence, spinal cord damage was not observed. Overall, no significant difference in acute toxicities or late complications was found between these two groups (Table 4).

**Table 4:** Treatment toxicity for the two group (NBT+EBRT and EBRT).

Characteristics	NBT+EBRT, N (%)	EBRT N (%)	*p* value
**Acute toxicity**			
Esophagitis (RTOG)			0.427
Grade 0–1	8(26)	14(45)	
Grade 2–3	23 (74)	17 (55)	
Grade 4	0	0	
Pulmonary complications(RTOG)			0.341
Grade 0–1	29(93.6)	27(87.1)	
Grade 2–3	2 (6.4)	4 (12.9)	
Grade 4	0	0	
Hematologic toxicities(WHO)			0.649
Grade 0–1	17(55)	18(58)	
Grade 2–3	14 (45)	13 (42)	
Grade 4	0	0	
**Late toxicity**			0.537
Esophageal fistulas	2 (6.45)	2 (6.45)	
Massive bleeding	3 (3.2)	2(6.45)	

*EBRT* external beam radiotherapy, *NBT* neutron intraluminal brachytherapy.

## Discussion

Definitive CRT or RT is a treatment alternative for patients with unresectable advanced cancer or patients who refuse surgery. However, merely 30%-62% of patients who receive CRT achieve pathological complete response. More importantly, the recurrence rate of this approach is high. Esophageal carcinomas are often metachronous or occur with multiple malignancies^11^. Recurrence in esophageal carcinomas remains a major challenge. Studies have reported that for surgical approaches, the local recurrence rate is 12.1%, and the incidence of lymph node metastasis is 18.2%^12^. However, the local recurrence rate after RT/CRT could reach as high as 78.4%, with a 33.3% recurrence rate of lymph node metastasis^13^. The method, which depends on the anatomical location of the recurrent lesion, initial treatment strategy and tumor response, influences salvage treatment. The optimal salvage treatment remains to be elucidated. For patients with local failure after CRT, salvage surgery has been suggested. However, the rates of complications and mortality are high. Furthermore, salvage surgery is not recommended for patients with locally advanced, nonresectable, or inoperable tumors^14–17^. Some papers have reported that EBRT+NBT can improve PFS and OS in patients with advanced cervical and esophageal carcinomas^18,19^. Zhi-guo Zhou^20^ reported that compared to chemotherapy, gastrostomy and stent implantation, reirradiation could improve OS in patients with local recurrence of esophageal carcinoma after definitive RT or chemoradiotherapy (CRT). The present retrospective analysis revealed that using NBT as an adjuvant treatment for recurrent esophageal cancer in conjunction with EBRT was effective and well tolerated by patients. This treatment approach improved local control because neutrons are more effective than photons in killing radioresistant tumor cells and do not increase the rate of late and severe complications. However, there was no significant improvement in OS. Two reasons may explain these results. The first may be due to the highly similar irradiation dose. The second may be due to the relatively small cohort.

The RTOG9405/INT 0123 trial reported that compared to the standard irradiation dose for esophageal carcinoma (50.4 Gy), the higher dose (64.8 Gy) did not improve survival or local control^21^. However, other papers reported that the standard irradiation dose of approximately 60 Gy for the recurrence of esophageal carcinoma following surgery could improve survival^22^. Taggar et al.^23^ reported that high brachytherapy doses may improve overall survival. Therefore, with a high reirradiation dose in our cohort, the dose of EBRT was 54–60 Gy/30 f., and that of NBT+EBRT was 12 Gy-eq/3 f. + 41.4 Gy/23 f. or 12 Gy-eq/3 f. + 46 Gy/23 f.

Consistent with previous studies, recurrence markedly shortened the survival duration. The median OS duration was 19 months in the nonrecurrence group and 9.25 months in the recurrence group. Notably, more than 45% of patients relapsed within one year after irradiation, with a 1-year local control rate of 64.5%. Ishihara et al. revealed similar findings, in which 82% of recurrences developed within 21 months of CRT^24^. The 1-, 2- and 3-year OS rates of recurrent patients who received salvage RT were 83.8%, 37% and 8%, respectively, which were promising when compared with those reported in other studies^25^. Yamashita et al.^26^ reported that curative surgery on the locoregional recurrence of esophageal cancer yielded a median survival time of 13.8 months and a 1-year survival rate of 56%. Furthermore, Jingu et al*.* reported that 5-fluorouracil concurrent with CRT yielded a 3-year survival rate of 56.3%^27^. Nicolay et al. reported that salvage high-dose-rate brachytherapy for esophageal cancer in previously irradiated patients yielded a median local PFS duration of 9.8 months and 1- and 2-year survival rates of 31.5% and 17.5%, respectively^28^. Last, Amandeep S Taggar et al. reported that endoluminal high-dose-rate brachytherapy for locally recurrent or persistent esophageal cancer yielded a median survival time of 20.9 months and a 1-year survival rate of 78%^23^. The variation in these findings might be attributed to the conditions at the time of treatment, the locations of recurrent lesions and alternative therapies received by patients. The survival rate was low in patients who experienced recurrence at or within one year after radical RT/CRT. The growth rate of recurrent tumors is likely associated with the time of recurrence. In particular, early recurrences may arise from fast-growing or hypoxic and therapy-resistant tumor cells. Compared with X-ray, californium-252 neutron brachytherapy has a high-LET nature, making it much more effective in killing hypoxic and therapy-resistant tumor cells. So in our study the EBRT+NBT may be suited to patients with short-term recrudescence or the nearby normal tissues dose must be low, and received a significantly lower IMRT(41.4–46 Gy, four fractions/week, one fraction/day, and 1.8–2.0 Gy/fraction, for total of 23 fractions), this can be addressed by reference to the additional dose from the NBT. We will further study the effect of Californium-252 neutron brachytherapy in patients with early recurrence.

In the present study, the main causes of death were different between the NBT+EBRT group and the EBRT group. The NBT+EBRT group had metastasis, and the EBRT group had local regional recurrence. It was posited that high linear energy transfer ^252^Cf-NBT is superior to conventional RT (X-ray) for esophageal cancers, which are generally radioresistant or hypoxic. A neutron dose to nearby normal tissues can be reduced by water injection into the source applicator. Salvage RT was completed without radiation myelitis or spinal cord damage in all patients in the NBT+EBRT group, especially in patients with short-term recrudescence or those in whom nearby normal tissues doses had to be low to tolerate therapy. However, there was no significant difference in the rate of acute toxicities or late complications between these two groups.

## Conclusion

The limitations of the present study were its retrospective nature and the relatively small cohort. However, NBT+EBRT for recurrent esophageal carcinoma after CRT is an effective treatment option, and it is especially suitable for patients with short-term recrudescence. Patients who were treated with ^252^Cf-NBT in combination with EBRT achieved better local control.

## Abbreviations

^252^Cf: Californium-252
ARS: After-recurrence survival
CR: Complete response
CRT: Chemoradiotherapy
CT: Computed tomography
CTV: Clinical target volume
EBRT: External beam radiotherapy
GTV: Gross tumor volume
NBT: Neutron intraluminal brachytherapy
PR: Partial response
PTV: Planning target volume
RFS: Recurrence-free survival
RT: Radiotherapy
SD: Stable disease
RBE: Relative biological effectiveness
